# Multilingualism, multicultural experience, cognition, and creativity

**DOI:** 10.3389/fpsyg.2023.1155158

**Published:** 2023-11-09

**Authors:** Guillaume Fürst, François Grin

**Affiliations:** ^1^Faculty of Translation and Interpreting, University of Geneva, Geneva, Switzerland; ^2^Faculty of Psychology, Swiss Distance Learning University, Brig, Switzerland; ^3^CIRANO, Montréal, QC, Canada

**Keywords:** creativity, cognition, multilingualism, multicultural experience, structural model

## Abstract

The once widely held notion that bilingualism is related to enhanced cognitive functions has recently been challenged, in particular among young adults, as opposed to children and older adults. This strand of research, however, is essentially focused on executive functions (e.g., attention, inhibition, and shifting). But there is another side to the bilingualism-cognition story. Indeed, growing evidence has shown that bilingualism, and by extension multilingualism, are associated with enhanced creativity. However, this relation is arguably quite complex, for several reasons. First, creativity is a fuzzy notion; it is usually conceptualized as a mix of cognitive, personality and motivational factors. Second, multilingual people generally have a richer multicultural experience than monolingual people. In addition, multicultural experience itself is also positively related to creativity. Hence, there are manifold relations between cognition, creativity, multilingualism, and multicultural experience. In this brief research report, using a latent variables model which replicates some of our recent findings, we show that both multilingualism and multicultural experience are positively associated with creativity, even when controlling for cognitive abilities (divergent thinking and intelligence). We discuss these results in a perspective that considers methodological challenges and factors that are relevant to goal-directed behavior.

## 1. Introduction

The “bilingual advantage,” the idea according to which bilingualism strengthens executive functions (e.g., Bialystok, 2009; Bialystok et al., 2012), has been challenged recently. In particular, a recent meta-analysis has questioned the robustness and generality of this association in adults (Lehtonen et al., 2018). This meta-analysis reported “a very small bilingual advantage for inhibition, shifting, and working memory” (p. 394), an advantage that disappears when correcting for publication bias, hence suggesting that there is actually no systematic association between bilingualism and executive functions. This view was reinforced by Nichols et al. (2020), whose study, based on more than 10,000 participants, tested the link between bilingualism and executive functions, measured by 12 different tests. The authors found only negligible differences between bilinguals and monolinguals, and once again, these differences vanished when potentially confounding factors were taken into account (e.g., gender, age, level of education, and socio-economic status). Given these recent results, it appears that the “bilingual advantage” is very thin to inexistent, at least in adults. However, the debate remains open and ongoing. The main points of contention are that (1) executive functions are a complex and heterogeneous set of processes which is difficult to assess as a whole, and (2) the cognitive advantage of bilingualism in children ought to be more consistently observed (Paap, 2019).

A recent review of the impact of bilingualism on executive functions among children and adolescents (Giovannoli et al., 2020) partially supports this view, showing that there is indeed evidence suggesting a positive impact of bilingualism on inhibition and cognitive flexibility, but not on working memory. Grundy (2020), using a Bayesian analysis, concludes that “when effects do appear, they tend to favor bilinguals outperforming monolinguals” (p. 190) and that there is a need to clarify *when* (rather than *if*) bilinguals outperform monolinguals. In addition, it also appears that in older adults, bilingualism contributes to cognitive reserve and is associated with a delay in the onset of symptoms of dementia (Bialystok et al., 2012; Bialystok, 2017). However, Paap (2019) also mentions that several methodological and statistical issues plague the field, in particular potential confound variables (e.g., socio-economic status, intelligence, culture, and immigrant status).

Another, related and more recent strand of research has argued that there is a positive correlation between creativity and multilingualism (e.g., Kharkhurin, 2012). This research tradition is much younger than, and nowhere as systematic as research on bilingualism and executive functions. However, the “creativity and multilingualism” hypothesis has received some empirical support. For example, Kharkhurin has shown that Russian-English bilingual immigrants performed better on verbal divergent thinking tasks than monolingual native speakers (Kharkhurin, 2008) and that Farsi-English bilinguals had a higher capacity for innovation than monolinguals, as measured by originality of ideas, both in a verbal divergent thinking task and in a drawing task (Kharkhurin, 2009). In another study, however, Kharkhurin (2010) found that Russian-English bilinguals had higher creativity scores in non-verbal divergent thinking tasks but lower creativity scores in verbal tasks. Moreover, recent studies have provided evidence against a link between bilingualism and creativity. In a pre-registered study, Booton et al. (2021) have found no difference between bilingual and monolingual children in a variety of divergent thinking tasks. Lange et al. (2020) found similar results—showing no effect—in young adults. Therefore, overall, results are mixed.

Regarding the possible mechanisms explaining the link between bilingualism and creativity, some work has suggested the positive role of conceptual complexity, inhibition, selective attention, or code-switching habits (Kharkhurin, 2011, 2017; Kharkhurin and Wei, 2015). However, none of these explanations has received strong empirical support so far, and some contradictory work exists. The studies that did find a connection between bilingualism, creativity and selective attention (Kharkhurin, 2011; Xia et al., 2022) compared linguistically advanced students with less advanced ones, hence the effects of interest may be contaminated by a general positive effect of intelligence or personality. Kharkhurin and Wei (2015) also reported that selective attention was not related to code-switching habits; so even if selective attention is related to creativity, it is not clear how it connects to multilingualism, except through the (controversial) general effect of enhanced executive functions. Lange et al. (2020) have shown that the complexity of semantic network structure were not a likely explanation either. Finally, creativity has also been found to be associated not only with inhibition but also with disinhibition (e.g., Eysenck, 1995; Martindale, 2007; Carson, 2014).

Another recurring limitation of this research, however, is that most studies focus on immigrant populations, who also tend to have a strong bicultural experience. This positive correlation between bilingualism and bicultural experience is of critical importance, since bicultural experience has also been found to be positively correlated with creativity. For instance, Maddux and Galinsky (2009) have shown that time spent abroad has a positive impact on creativity. Other studies have suggested that individuals who identify with two cultures demonstrate greater creativity than assimilated or marginalized individuals who identify with only one culture (Benet-Martínez et al., 2006; Tadmor et al., 2012). Along these lines, Gocłowska and Crisp (2014) proposed that dual-identity processes foster creativity, arguing that individuals with complex social identities need to alternate their identities across contexts, as well as to integrate distant and potentially conflicting cultural elements. According to this approach, these processes are in turn related to enhanced cognitive flexibility and greater ease in integrating distant and conflicting ideas.

In short, there are complex and multiple links between creativity, multilingualism, and diversity of cultural experience. Accordingly, in our previous research (Fürst and Grin, 2018b, 2021), we have insisted on the need to consider simultaneously the effect of cultural experience and language skills on creativity. We have also proposed moving beyond strict bilingualism and biculturalism, and beyond the focus on immigrant populations, in order to use a broader conception of multilingualism and multicultural experience. Empirically, we have measured skills levels in various foreign languages (up to three) as well as experience such as traveling and living abroad.^1^ In line with many theoretical approaches of creativity (e.g., Sternberg, 1999; Kaufman and Sternberg, 2010, 2019), we have also insisted on the fact that creativity is a complex phenomenon (related to cognition, personality, affect, etc.) that calls for a multivariate approach. We found that multilingualism was related to various aspects of creative personality (e.g., openness, idea generation) and to various creative activities and achievements, including creative performance in creativity tasks. Multicultural experience was also found to be a significant predictor of several creativity variables. The general pattern, therefore, was that multilingualism and multicultural experience were mostly complementary—and not redundant—when predicting creativity. In the present study, we propose a replication of some of these previous results, including some cognitive variables as additional controls (divergent thinking and general intelligence).

## 2. Methods

The data presented here were collected as part of a larger study on both individual and group creativity. In what follows, we focus on individual creativity data only. The total sample was gathered in two successive waves, the first in winter 2020–2021 and the second in spring 2022. However, for the individual side of the study discussed in this paper, there are no relevant differences between the first and the second wave. Hence, in what follows, we describe the full sample directly—and, by extension, we perform all the analyses on the full sample.

### 2.1. Participants and procedure

The total sample size is *n* = 336. Of these participants, 76.6% were female. The mean age was 21.8 years old (min. = 18, max. = 38, SD = 3.1). Most participants (82.1%) were enrolled in BA programs; a minority were enrolled in MA programs (15.6%) or other degrees (1.5%). Participants were studying in different faculties or schools of the University of Geneva; the faculties most represented were Translation and Interpreting (17.4%), Law (13.2%), Economics and Management (12.6%), Social Sciences (11.8%), and Psychology and Educational Sciences (11.8%).

All the data collection took place online. Participants were recruited on the University of Geneva campus using posters and flyers, as well as through announcements in various courses. Participants registered for the study by email and received a link to an online questionnaire. All questionnaires and tasks were presented in fixed order, the same for all participants—first multilingualism and multicultural experience questionnaires, then personality and creativity questionnaires, and finally cognitive tasks. As part of the larger study, participants also registered for a one-hour group creativity session that took place on Zoom (not discussed here). This other phase occurred several days after the data discussed in the present paper were gathered. Participants received a financial compensation of 30 CHF (about USD 30) for a total participation time of about 90 min.

### 2.2. Measures

Four main categories of variables were assessed in this study: cognitive abilities, creativity, multilingualism, and multicultural experience.

#### 2.2.1. Cognitive abilities

Divergent thinking abilities were assessed with two classical tasks: “name all the uses of a cardboard box you can think of” (Torrance, 1966) and “name all the round things you can think of” (Wallach and Kogan, 1965). Both of these tasks were time-limited to 3 min each. In the analyses below, we only use the fluency scores obtained in these tasks. On average, participants gave more ideas in the second task (M = 12.42; SD = 4.48) that in the first task (M = 7.43; SD = 3.09).

We also assessed general intelligence with the 12 items of Raven’s Advanced Progressive Matrices Set I (Raven et al., 2003). We used this set because it was an adequate short intelligence test of medium difficulty for this sample—given that it is entirely composed of university students, the Standard Progressive Matrices would have been too easy. We used the first item as an example; scores can therefore vary between 0 and 11. Descriptive statistics show that 12.8% of participant have reach the maximum score (M = 8.69; SD = 1.73).

#### 2.2.2. Creativity

##### 2.2.2.1. Creative personality

We conceptualized creative personality using three complementary variables: Openness, Intellect, and Idea Generation. For all of the personality items, describing various behaviors or habits, participants answered using a five-point scale, from 1 = “almost never” to 5 = “very often.” Openness (interest in esthetics, fantasy, and imagination) and Intellect (interest in ideas and intellectual activities) were assessed using the scales developed by DeYoung et al. (2007). In their original versions, these were two 10-item scales; in the present study, we used shorter versions, with six items for each scale (see also Fürst and Grin, 2018a). In the present data set, the reliability of both scales was acceptable, with Cronbach’s alpha at 0.74 for intellect and 0.69 for openness. Idea Generation was measured using six items (see also Fürst and Grin, 2018a). Examples of items are “I easily come up with a lot of ideas”; “I like to play with ideas just for fun.” The reliability of the Generation scale was good, with a Cronbach’s alpha of 0.86.

##### 2.2.2.2. Creative interests, activities, and achievements

Creative interests, activities, and accomplishments were assessed in seven broad domains (or groups of domains) using a scale also used in our previous research (Fürst and Grin, 2018a). These domains are: music (singing, musical instrument, and composition); literature/writing (fiction, prose, and screenplays); performing arts (dance, theater, and comedy), visual arts (drawing, photography, and graphic design), 3D design (architecture, industrial design, and fashion), inventions and technical solutions (DIY, electronics, and computer programming), and science (academic work in the humanities or life sciences).

For each of these domains, an initial yes/no question was asked to ascertain whether participants had any interest in the domain concerned. If they answered “yes,” six more questions were asked about the intensity of that interest (e.g., “I am interested in many things related to this area”; “I like to learn new things in this area”). Responses to these questions used a scale ranging from 1 = “almost never” to 5 = “very often.” The sum of these six items across the seven domains gives the creative interest variable. Virtually all participants (98.5%) had at least minimal interest in one domain. The corresponding Cronbach’s alphas for these six items in the seven domains ranged from 0.77 to 0.87.

Another yes/no question asked whether participants had (or had had) active practice in a given area. If a participant answered “yes,” two groups of five additional questions were asked. The first group of questions concerned the intensity of participation in an activity. Example of items for an activity include “I spend several hours a week practicing in this area,” “My practice in this area is significant and important to me.” Responses to these questions were given on a scale from 1 = “almost never” to 5 = “very often.” The sum of these five items across the seven domains yields the creative activities variable. The corresponding Cronbach’s alphas for these six items in the seven domains are all greater than 0.78, with the exception of the “invention” domain, which has a lower fidelity (0.46).

The second group of questions focused on creative accomplishments. Sample items for this domain include “I have won awards or prizes in this field”; “I have been paid for my work in this field.” Responses were scored using the following scale: 1 = “never”; 2 = “once or twice”; 3 = “between 3 and 5 times”; 4 = “6–10 times”; 5 = “11 times or more.” The sum of these five items in the seven domains yields the creative achievement variable. The corresponding Cronbach’s alphas for these six items in the seven domains are all greater than 0.68, with the exception of the “invention” domain, which has a very low fidelity (0.10). Despite the low fidelity of this domain, we decided to keep it in the total scores (analyses with and without it yielded the same results).

#### 2.2.3. Multilingualism

We measured language skills in three foreign languages (L2, L3, and L4). For each of these languages, four competences were assessed (oral comprehension, speaking, reading, and writing) using a self-reporting matrix of language tasks based on the Common European Framework of Reference for Languages (CEFRL), which benefits from a substantial body of research on the meaning and measurement of language skills (Council of Europe, 2020). For each skill in each language declared, the possible scores are “1” (traces of elementary skills), “2” (equivalent to CEFR level A1), “3” (equivalent to level A2), “4” (equivalent to level B1), “5” (equivalent to level B2), “6” (equivalent to level C1), and “7” (equivalent to level C2). The self-reporting questionnaire uses objective descriptors, for example “I can introduce someone and use simple greetings and expressions” [A1 level] or “I can express my opinion on topics that are familiar to me” [B1 level], etc. These descriptors have also been used and have performed well in another study with nearly 50,000 participants (Grin et al., 2015).

These data allowed us to construct three language skills variables: overall skills in L2, overall skills in L3, and overall skills in L4. For each language (L2, L3, and L4), we constructed a total score (mean of all competences), that can vary between 1 and 7. For participants who have declared no skills whatsoever in a given foreign language (for example L4), we assigned a value of 0. In this data set, all participants declared some competences in a second language (L2; M = 5.8; SD = 1.12), 93.1% declared some competences in a third language (L3; M = 4.51; SD = 1.94), and 45.6% declared some competences in a fourth language (L4; M = 2.22; SD = 2.3).

#### 2.2.4. Multicultural experience

We used three indicators to capture multicultural experience. The first was simply the number of countries in which participants have lived (scale from 1 = “I’ve always lived in the same country” to 5 = “five or more different countries”). About half of the participants (46.7%) has always lived in the same country and only a minority (8.1%) has lived in more than three countries.

The two other indicators were constructed from a question about “important countries.” We introduce this concept to participants as

The countries that matter to you. By this we mean, for example, your current country of residence, the country of origin of your family, a country where you have lived or traveled for a long time and/or several times, a country where you have had an important life experience, a country that has made an impression on you or that you feel close to for other reasons as well (e.g., a taste for the culture, music, or literature of that country).

Participants could list up to five important countries (“number of important countries” variable). The mean value of this variable is 3.4 (SD = 1.15). This variable was initially used in Fürst and Grin (2018b). In addition, for each country, participants were asked how important a given country was, using a scale of 1–5, where 1 was “not very important” and 5 was “really very important.” The sum of these ratings generates the variable “total rating of important countries” (M = 13.55; SD = 4.58). This variable is new and unique to the present study.

### 2.3. Data analysis

**Figure 1:**
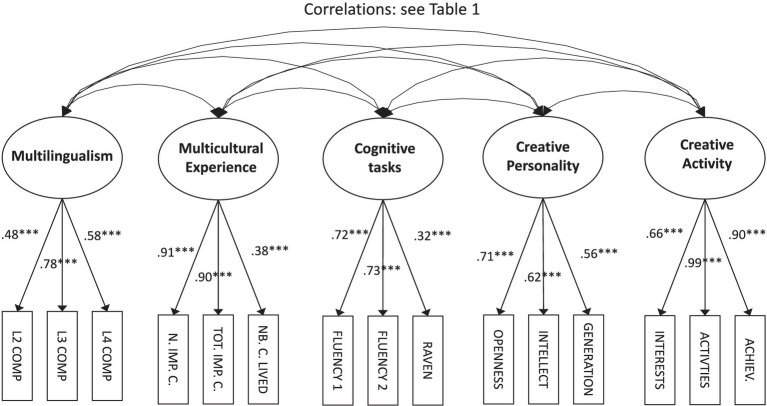
Model 1: correlations between all variables. ^***^p < 0.001. All parameters are standardized. L2 COMP, Competences in second language; L3 COMP, Competences in third language; L4 COMP, Competences in fourth language; IMP. C., Number of important countries; TOT. IMP. C., Total rating of important countries; NB. C. LIVED, Number of countries in which participants have lived; FLUENCY 1, Fluency in “box” task; FLUENCY 2, Fluency in “round” task; RAVEN, Raven’s Advanced Progressive Matrices; INTERESTS, Creative interests; ACTIVITIES, Creative activities; and ACHIEV., Creative achievements.

**Figure 2:**
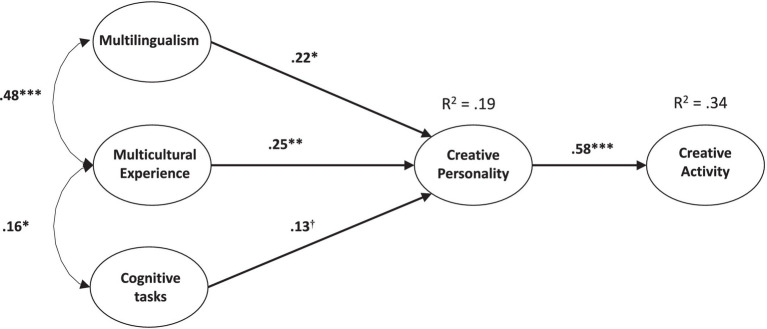
Model 2: multiple regression and mediation. ^†^*p* < 0.10; ^*^p < 0.05; ^**^p < 0.01; ^***^p < 0.001. All parameters are standardized.

We performed data analysis using a latent variable approach (structural equation models) using Mplus 6 (Muthén and Muthén, 2007). Specifically, we constructed the following latent variable: *Multilingualism* (competences in L2, L3, and L4); *Multicultural Experience* (number of important countries, total rating of importance, and number of countries in which one has lived); *Cognitive tasks* (two fluency scores, Raven’s matrices); *Creative Personality* (openness, intellect, and idea generation),^2^ and *Creative Activities* (creative interests, activities, and achievements). On this basis, we estimated a first model, in which all correlations between variables were estimated (Figure 1). Then we estimated a more parsimonious model, a multiple regression and mediation model (Figure 2).

To assess model fit, we used the Root mean square error of approximation (RMSEA) and its 95% confidence interval, the standardized root mean square residual (SRMR), and the comparative fit index (CFI). Generally speaking, one can say that a model has a good fit if CFI is around 0.95 or higher, SRMR around 0.08 or lower, and RMSEA around 0.06 or lower (Hu and Bentler, 1999; Iacobucci, 2010). Our models, whose fit indices are presented below, had an overall satisfactory fit, with very good SRMR values and acceptable RMSEA and CFI values. More importantly, all factor loadings were significant and generally strong; the relation between latent variables was informative and meaningful.

## 3. Results

Our first model is depicted in Figure 1; correlations between all factors of this model are provided in Table 1. As mentioned above, the fit of this model is satisfactory, with *χ^2^*(80) = 231.3, *p* < 0.01, RMSEA = 0.075 (IC_95%_ [0.064; 0.086]), SRMR = 0.057, and CFI = 0.92. Most factor loadings are higher than 0.70 and very few are below 0.40. One exception is the “Raven” indicator of the *Cognitive tasks* latent variable, with a loading of 0.32. This indicates that this latent variable is more strongly defined by divergent thinking abilities (both fluency scores have loadings above 0.70). The other exception is the indicator “number of countries in which one has lived” of the *Multicultural Experience* factor, with a loading of 0.38. This indicates that this factor is essentially defined by the two “important countries” variables—that is, the number of important countries listed by the participants and the total score of importance assigned to these countries.

**Table 1:** Correlations between latent variables.

	ML	MCE	COG	CP	CA
Multilingualism (ML)	-				
Multicultural experience (MCE)	0.48^***^	-			
Cognitive tasks (COG)	−0.07	0.14^*^	-		
Creative personality (CP)	0.36^***^	0.38^***^	0.16^*^	-	
Creative activity (CA)	0.14^*^	0.22^***^	0.07	0.59^***^	-

^*^p < 0.05; ^***^p < 0.001.

The correlations between factors described in Table 1 are results of central interest here. As expected, we first note a strong correlation between Multilingualism and Multicultural Experience (*r* = 0.48). The correlation between the two latent creativity variables is also high (*r* = 0.59). Multilingualism is significantly associated with both Creative Personality and Creative Activity (*r* = 0.34 and *r* = 0.14, respectively). Multicultural Experience is also significantly associated with these two latent creativity variables (*r* = 0.38 and *r* = 0.22, respectively). The latent Cognitive tasks variable is positively correlated to Creative Personality (*r* = 0.14) and to Multicultural Experience (*r* = 0.14). Finally, there is no significant correlation between Multilingualism and Cognitive tasks, nor between Cognitive tasks and Creative Activity.

Noting that some correlations were weak (e.g., between Multilingualism and Creative Activity), we decided to test a second, more parsimonious model. More fundamentally, the chief motivation with model 2 was to propose a simpler pattern of relations than the full correlation matrix between all factors (described in Table 1). Specifically, we wanted to test whether multilingualism and multicultural experience were still associated with creative activity after controlling for creative personality, which is obviously the stronger predictor of creative activity.

In this model, depicted in Figure 2, Creative Activity is predicted only by Creative Personality, which is in turn predicted by Multilingualism, Multicultural Experience, and Cognitive tasks. This model is more parsimonious because four relations are set to 0: between Creative Activity and Multilingualism, between Creative Activity and Multicultural Experience, between Creative Activity and Cognitive tasks, and between Multilingualism and Cognitive tasks. The fit of this model is virtually the same as that of model 1: *χ^2^*(84) = 234, *p* < 0.01, RMSEA = 0.073 (IC_95%_ [0.062; 0.084]), SRMR = 0.058, and CFI = 0.92. The parameters that have been set to 0 are non-significant when estimated.

In other words, this means that there is no direct effect of Multilingualism, Multicultural Experience, and Cognitive tasks on Creative Activity; all the key relations between the variables at hand are represented in this more parsimonious model. First, we find again the strong relation between Creative Activity and Creative Personality (*β* = 0.58), as well as between Multilingualism and Multicultural Experience (*r* = 0.48). Then, we see that Multilingualism, Multicultural Experience, and Cognitive tasks all predict a part of the variance in Creative Personality (*β_s_* = 0.22, 0.25, 0.13, respectively). The *β_s_* for Multilingualism and Multicultural Experience are lower than the *r_s_* reported in Table 1, indicating a certain level of convergence in their predictive roles, but the fact that both of them remain significant also indicates complementarity between them.

## 4. Discussion

Overall, the results discussed here replicate what we found in previous studies (Fürst and Grin, 2018b, 2021). One of the key findings is that multilingualism and multicultural experience both contribute to creativity. Although these two variables are quite strongly correlated, they are not redundant when predicting creativity, as shown in Model 2. The strengths of these relations are modest (in the 0.20–0.30 range), but they seem robust—not only here but also across previous studies.

The data and modeling strategy presented here go beyond earlier research. First, the present study includes measurements of cognitive abilities.^3^ As mentioned in the results section, the cognition factor considered here is essentially represented by divergent thinking abilities (fluency) and, to a lesser extent, by general intelligence. Hence, unsurprisingly, we found that this factor was positively related to the creative personality factor. The link between creative personality (or openness) and divergent thinking is fairly commonplace and widely documented in previous literature (e.g., McCrae, 1987; Fürst and Grin, 2018a).

We found no significant correlation between multilingualism and intelligence—neither between the latent variables presented here, nor at the level of single indicators, for instance between L2, L3, or L4 skills and the Raven’s Progressive Matrices. This result, however, is not entirely at odds with the previous literature, which, although quite limited, tends to show rather weak correlations between intelligence and foreign language acquisition (Salehi and Sadighi, 2012). Indeed, it seems that many other factors—motivation, personality, and learning opportunities—are more strongly associated with language acquisition (Khasinah, 2014; Grin et al., 2015). More generally, a recent study by Bialystok et al. (2022) has also shown than there is no difference in nonverbal intelligence between bilinguals and monolinguals.

Also quite unexpectedly, we found a positive correlation between cognitive tasks and multicultural experience. Additional correlation analyses show that multicultural experience is actually only correlated with fluency scores (see Appendix I), and not with Raven’s Progressive Matrices. This result is consistent with the idea that complex identity (emotional attachment to several countries) is associated with higher flexibility and the widening of one’s creative idea base (Gocłowska and Crisp, 2014). However, this result may also be related to the fact that the “important countries” variable is based on an enumeration, just as divergent thinking tasks are.

Additionally, Model 2 also reveals that, among the predictors available in this study, nothing predicts creative activities better than creative personality does. Indeed, the other variables—cognitive tasks, multilingualism, and multicultural experience—added nothing to the prediction of creative activities once creative personality was considered. We believe that this way of modeling the relation between the variables at hand and the corresponding results is important: it allows an important simplification to the potentially complex web of possible relations between all these variables.^4^

Therefore, the key variable here is creative personality, which immediately raises difficult questions about the direction of causality. Openness is a key component of creative personality. Open people are more likely to be creative. They are also generally more open to the world, to foreign languages, and to other cultures. Curiosity in the broadest sense, from intellectual curiosity to the search for new experiences, is at the heart of creativity, exploration, and learning. Thus, the causality behind the positive links between creative personality, multilingualism and multicultural experience remains unclear at this point. Does multicultural experience facilitate language acquisition, or *vice-versa*? Does openness promote multicultural experience and language learning, or does causality flow in the opposite direction? Or both? Only a large-scale, longitudinal study could provide precise answers to these questions. To the best of our knowledge, no such studies exist.

In this context, we believe that great caution is required when it comes to concluding on the nature of the link between creativity and multicultural experience or multilingualism. The lack of longitudinal studies is not the only reason for such caution. First, creativity, like executive functions (and even more so) is a fuzzy concept, and one which it is difficult to pin down. Strictly speaking, the only positive results we found so far were obtained with a relatively general questionnaire of creativity and with a few quite specific creativity tasks (Fürst and Grin, 2018b, 2021). Let us recall that all these tasks were verbal in nature (a short story writing task or a remote association task). In contrast with Kharkhurin (2009), we did not find a positive correlation between non-verbal creativity and multilingualism. In contrast with Maddux and Galinsky (2009), we did not find a positive relation between remote association abilities and experience of living abroad—but instead, we find an effect of multilingualism on these abilities.

In this paper, although no complex or real-life creativity tasks are included (creative activities and achievements rely only on self-report), Model 2 strongly suggests that most of the link between creativity and multicultural experience or multilingualism boils down to the creative personality and openness factors. Furthermore, in this study (as in our previous studies), there was no probable serious confound variable. All participants were university students, thus having a relatively homogenous level of intelligence and socio-economic status. No substantial part of the sample was made up of immigrants—whether privileged or not—and no specific cultural minority was particularly predominant.

Overall, we believe that our approach, by focusing on multilingualism (instead of just bilingualism) and multicultural experience (instead of more specific bicultural identity) both opens and strengthens the research on the links between diversity and creativity. Still, the approach could be even more extensive. First, it might be interesting to add measures checking whether people routinely engage in spontaneous code-mixing and code-switching between their languages (independently of the level of competence). Such measures may be based on the frequency and variety of situations in which various languages are used (e.g., Grin et al., 2015) or on questionnaires directly assessing code-mixing and code-switching habits (e.g., Low and Lu, 2006; Byers-Heinlein, 2013). Similar extensions could be considered for multicultural experience, since, for instance, people can be exposed to different cultures or sub-cultures within a specific country. Although the concept of culture or subculture is extremely difficult to operationalize satisfactorily, questionnaires assessing multicultural experience could be used (Aytug et al., 2018). Further research is also necessary to assess the validity and strength of the causal direction generally assumed, which flows from linguistic and/or cultural diversity to creativity. One strategy is to use longitudinal designs and cross-lagged panel models. Another is an instrumental variable approach with an instrument that generates exogenous variation in multilingual skills and/or multicultural experience, but not in key independent variables, such as openness, which are linked to both multilingualism and multiculturalism on the one hand, and creativity on the other hand. A third approach, of course, is to use suitable experimental designs (e.g., experimental situations that mimic multicultural exposure, since it is hardly possible to assign people randomly to real cultures or languages). Such approaches have already been used (Maddux and Galinsky, 2009; Leung and Chiu, 2010) and it may be worthwhile to deepen them.

If the outcomes of such analyses end up reinforcing the likelihood of a causal link from diversity to creativity, this would, in turn, strengthen the case for viewing multilingual skills and multicultural experience as relevant to goal-directed behavior. Examples can be found at three levels, micro, meso, and macro. At the micro level of individual actors, foreign language learning, as well as exposure (e.g., through international travel) to a broad range of cultures may be interpreted as a relevant strategy for enhancing individual creativity, which may give rise to the wide range of favorable effects generally associated with it, for example in the work sphere. At the meso level of organizations, first and foremost private-sector companies but also in the civil service, the widely-held notion that diverse teams are more creative would then be placed on a much firmer footing. At the macro level of societies, establishing this causal link would constitute a powerful argument for encouraging foreign language learning and intercultural contact, which would make for more creative, and hence more vibrant and resilient societies.

## Data availability statement

The original contributions presented in the study are included in the article/Supplementary material, and further inquiries can be directed to the corresponding author.

## Ethics statement

Ethical approval was not required because according to the University of Geneva’s official guidelines applying at the time of project submission and validation, no formal approval by an ethics committee was required given the non-sensitive character of the data. The studies were conducted in accordance with the local legislation and institutional requirements. Written informed consent for participation was not required from the participants because participation was strictly voluntary, and participants were fully informed beforehand and in writing of the study’s goals and contents, as well as of the guarantee of complete anonymity in data collection and treatment.

## Author contributions

GF and FG have both contributed to the conception and design of the study and have jointly written the discussion. GF organized the database and performed the statistical analyses, and wrote the first draft of the manuscript. All authors contributed to the article and approved the submitted version.

## Footnotes

^1^The epistemological implications of this choice are addressed in the concluding section, in particular the possible avenues for broadening the conception and measurement of multilingualism.

^2^The Idea Generation variable was considered as a personality variable in this paper, although it can also be seen as a process or cognitive variable. On the one hand, some items clearly refer to cognitive processes (e.g., “I easily come up with a lot of ideas”); on the other hand, some items refer to interests and preferences (e.g., “I like to play with ideas”) as well as to behaviors such as exploration (e.g., “I like to experiment, explore new and unusual options”), which are clearly part of personality. Idea Generation is also quite similar to the “fantasy” facet of the Openness dimension of personality. Empirically, Idea Generation is also more strongly associated with Openness and Intellect than with cognitive tasks (See full correlation matrix between all variables in Appendix I).

^3^Of course, the factor “cognitive tasks” does not represent the full range of human cognition. Rather, it is only a sampling of selected abilities that are known to be relevant for creativity (fluency in particular, but also general intelligence). Our intention in this research was only to control for a few cognitive variables and not to provide a full-fledged analysis of the multiple relations between cognition, creativity, and multilingualism.

^4^Another way to describe these results would be to say that multilingualism and diversity of cultural experience have only indirect effects on creative activity. Those standardized effects are, respectively, 0.13 (*p* = 0.013) and 0.15 (*p* = 0.003).

## Appendix I: Full correlation matrix

**Table:**

	*Multilingualism*			*Multicultural experience*			*Cognitive tasks*			*Creative Personality*			*Creative Activities*		
	[1]	[2]	[3]	[4]	[5]	[6]	[7]	[8]	[9]	[10]	[11]	[12]	[13]	[14]	[15]
[1] L2 COMP	1														
[2] L3 COMP	**0.41**^**^	1													
[3] L4 COMP	**0.23**^**^	**0.44**^**^	1												
[4] NB. IMP. C.	0.16^**^	0.31^**^	0.30^**^	1											
[5] TOT. IMP. C.	0.14^**^	0.32^**^	0.35^**^	**0.83**^**^	1										
[6] NB. C. LIVED	0.29^**^	0.24^**^	0.26^**^	**0.35**^**^	**0.32**^**^	1									
[7] FLUENCY 1	−0.03	−0.06	−0.01	0.14^*^	0.10	−0.06	1								
[8] FLUENCY 2	0.00	−0.01	−0.07	0.12^*^	0.06	−0.13^*^	**0.53**^**^	1							
[9] RAVEN	0.04	0.00	−0.08	0.02	−0.07	−0.07	**0.23**^**^	**0.24**^**^	1						
[10] OPENESS	0.12^*^	0.20^**^	0.12^*^	0.28^**^	0.30^**^	0.14^*^	0.01	0.05	−0.04	1					
[11] INTELLECT	0.23^**^	0.22^**^	0.16^**^	0.17^**^	0.18^**^	0.18^**^	0.07	0.09	0.08	**0.41**^**^	1				
[12] GENERATION	0.05	0.10	0.05	0.12^*^	0.21^**^	0.04	0.17^**^	0.14^**^	0.01	**0.36**^**^	**0.45**^**^	1			
[13] INTERESTS	0.04	0.14^*^	0.08	0.17^**^	0.20^**^	0.05	0.01	0.07	0.07	0.55^**^	0.38^**^	0.28^**^	1		
[14] ACTIVITIES	0.04	0.11	0.09	0.20^**^	0.19^**^	0.08	0.01	0.08	0.06	0.47^**^	0.30^**^	0.28^**^	**0.65**^**^	1	
[15] ACHIEV.	0.13^*^	0.17^**^	0.10	0.23^**^	0.23^**^	0.15^**^	0.02	0.07	0.05	0.44^**^	0.26^**^	0.26^**^	**0.56**^**^	**0.89**^**^	1

^*^*p* < 0.05; ^**^*p* < 0.05; L2 COMP = competences in second language; L3 COMP, Competences in third language; L4 COMP, Competences in fourth language; IMP. C., Number of important countries; TOT. IMP. C., Total rating of important countries; NB. C. LIVED, Number of countries in which participants have lived; FLUENCY 1, Fluency in “box” task; FLUENCY 2, Fluency in “round” task; RAVEN, Raven’s advanced progressive matrices; INTERESTS, Creative interests; ACTIVITIES, Creative activities; ACHIEV., Creative achievements.
